# Tocilizumab modifies clinical and laboratory features of macrophage activation syndrome complicating systemic juvenile idiopathic arthritis

**DOI:** 10.1186/s12969-020-0399-1

**Published:** 2020-01-10

**Authors:** Masaki Shimizu, Mao Mizuta, Nami Okamoto, Takahiro Yasumi, Naomi Iwata, Hiroaki Umebayashi, Yuka Okura, Noriko Kinjo, Tomohiro Kubota, Yasuo Nakagishi, Kenichi Nishimura, Mariko Mohri, Masato Yashiro, Junko Yasumura, Hiroyuki Wakiguchi, Masaaki Mori

**Affiliations:** 1 Department of Pediatrics, Graduate School of Medical Sciences, Kanazawa University, 13-1 Takaramachi, Kanazawa, 920-8641 Japan; 2 Department of Pediatrics, Osaka Medical College, Takatsuki, Japan; 3 Department of Pediatrics, Kyoto University Graduate School of Medicine, Kyoto, Japan; 4 Department of Immunology and Infectious Diseases, Aichi Children’s Health and Medical Center, Obu, Japan; 5 Department of Rheumatics, Miyagi Children’s Hospital, Sendai, Japan; 6 Department of Pediatrics, KKR Sapporo Medical Center, Sapporo, Japan; 7 Department of Pediatrics, Faculty of Medicine, University of the Ryukyus, Nakagami-gun, Japan; 8 Department of Pediatrics, Graduate School of Medical and Dental Sciences, Kagoshima University, Kagoshima, Japan; 9 Department of Pediatric Rheumatology, Hyogo Prefectural Kobe Children’s Hospital, Kobe, Japan; 10 Department of Pediatrics, Yokohama City University Graduate School of Medicine, Yokohama, Japan; 11 Department of Lifetime Clinical Immunology, Graduate School of Medical and Dental Sciences, Tokyo Medical and Dental University, Tokyo, Japan; 12 Department of Pediatrics, Okayama University Hospital, Okayama, Japan; 13 Department of Pediatrics, Hiroshima University Graduate School of Biomedical & Health Sciences, Hiroshima, Japan; 14 Department of Pediatrics, Yamaguchi University Graduate School of Medicine, Ube, Japan

**Keywords:** Macrophage activation syndrome, Systemic juvenile idiopathic arthritis, Tocilizumab, Classification criteria

## Abstract

**Background:**

This study aimed to determine the influence of tocilizumab (TCZ) in modifying the clinical and laboratory features of macrophage activation syndrome (MAS) complicating systemic juvenile idiopathic arthritis (s-JIA). Furthermore, we assessed the performance of the 2016 MAS classification criteria for patients with s-JIA-associated MAS while treated with TCZ.

**Methods:**

A panel of 15 pediatric rheumatologists conducted a combination of expert consensus and analysis of real patient data. Clinical and laboratory features of s-JIA-associated MAS in 12 TCZ-treated patients and 18 untreated patients were evaluated. Possible MAS was defined as having characteristic laboratory features but lack of clinical features of MAS, or atypical MAS, or early treatment that prevented full-blown MAS.

**Results:**

Clinically, the TCZ-treated patients with s-JIA-associated MAS were less likely febrile and had significantly lower ferritin, triglyceride, and CRP levels than the untreated patients with s-JIA-associated MAS. Other laboratory features of MAS including lower platelet counts and lower fibrinogen were more pronounced in TCZ-treated patients. The TCZ-treated patients with s-JIA-associated MAS were less likely to be classified as MAS based on the MAS classification criteria (25% vs 83.3%, *p* < 0.01). This is ascribed to the absence of fever or insufficient ferritin elevation, compared with the untreated patients.

**Conclusion:**

TCZ could modify the clinical and laboratory features of s-JIA-associated MAS. When evaluating the s-JIA patients while treated with TCZ, it is not applicable to use MAS classification criteria. Care must be taken to not underdiagnose MAS based on the MAS classification criteria.

## Background

Macrophage activation syndrome (MAS) is a severe, potentially life-threatening complication of rheumatic diseases, which is clinically characterized by fever, hepatosplenomegaly, lymphadenopathy, profound depression of all three blood cell lines, deranged liver function, intravascular coagulation, and central nervous system dysfunction. The hallmark of MAS is an uncontrolled and dysfunctional immune response with excessive activation and expansion of T lymphocytes and macrophages exhibiting hemophagocytic activity, which leads to overproduction of numerous proinflammatory mediators, thereby eliciting a cytokine storm. MAS is complicated with many rheumatic diseases. However, MAS is most commonly seen in systemic juvenile idiopathic arthritis (s-JIA) and occurs in approximately 10% patients with s-JIA [1]. Furthermore, subclinical or occult MAS may occur in as many as 30–40% patients with active s-JIA [2].

s-JIA is a severe systemic inflammatory disorder of unknown etiology characterized by arthritis and systemic features such as spiking fever, skin rash, generalized lymphadenopathy, hepatosplenomegaly, and serositis. Although the pathophysiology of s-JIA remains obscure, it has been suggested that s-JIA is an auto-inflammatory condition driven by continuous activation of innate immune pathways, leading to aberrant induction of proinflammatory cytokines, such as interleukin (IL)-6, IL-1β, and IL-18 [1].

Tocilizumab (TCZ), a humanized anti-IL-6 receptor monoclonal antibody, serves as a clinically effective cytokine inhibitor for the treatment of s-JIA. Despite high efficacy for the treatment of s-JIA, recent studies have revealed that MAS could be complicated in patients with s-JIA receiving TCZ therapy [3, 4]. Furthermore, TCZ has been reported to modify and mask the clinical symptoms and laboratory findings of MAS in patients with s-JIA [3, 4].

On that account, prompt and rapid diagnosis is essential to initiate appropriate treatment for s-JIA-associated MAS. However, it is often difficult and challenging to distinguish s-JIA-associated MAS from s-JIA flares. The classification criteria for MAS in the setting of s-JIA based on expert consensus along with a large patient databank was reported in 2016 [5]. We previously validated these criteria and showed these criteria had a very high performance for the diagnosis of full-blown MAS with high sensitivity and specificity in the real world [6]. However, a recent systematic literature review revealed that the clinical and laboratory features of s-JIA-associated MAS could be modified in patients treated with biologic agents including TCZ [4].

Therefore, this study aimed to determine the influence of TCZ in modifying the clinical and laboratory features of s-JIA-associated MAS and to assess performance of the 2016 MAS classification criteria for patients with s-JIA-associated MAS while treated with TCZ in the real world.

## Methods

This is a multicenter, retrospective, case-control study including TCZ-treated and TCZ-untreated s-JIA patients with MAS. For patients with MAS, information on the laboratory features from acute s-JIA phase to MAS phase that included at least three time points (the last visit before MAS onset, the time of MAS onset, and the period of full-blown MAS) were collected. MAS onset was defined as the time when the initial clinical and/or laboratory abnormalities suggesting the occurrence of MAS were detected. Full-blown MAS was defined as the time at which MAS reached its most severe stage. Possible MAS was defined as the condition when patients had characteristic laboratory features but lack of clinical features of MAS or when early treatment prevented full-blown MAS [7, 8].

We enrolled 36 Japanese MAS patients treated with TCZ from 2006 to 2017. Investigators were asked to include s-JIA patients with MAS seen after 2006. This timeframe was chosen because preliminary diagnostic guidelines for the diagnosis of MAS was reported in 2005 [9]. We reported 36 patients from 14 institutes of pediatric rheumatology in Japan. A panel of 15 experts was first asked to diagnose the 36 patient profiles with or without MAS based on clinical and laboratory features at the time of disease onset. A combination of expert consensus and analysis of real patient data was conducted by a panel of 15 pediatric rheumatologists. The minimum required level of agreement among experts was set at 80%. Of the 36 patient profiles, 20 were excluded because of insufficient data. At the 36 patient data points for MAS, platelet counts and serum AST levels were measured in all 36 patient data points. On the other hand, serum ferritin levels were measured in 33 patient data points (91.6%), TG was measured in 23 patient data points (63.8%) and fibrinogen in 26 patient data points (72.2%). Among the remaining 16 patient profiles measured all items that fulfilled the MAS criteria, 12 were diagnosed with MAS and four without MAS by the experts. We also re-evaluated real patient data from 18 MAS patients not treated with TCZ in our previous historic cohort [6]. Clinical and laboratory features of s-JIA-associated MAS upon MAS diagnosis were evaluated, and the data was compared between the TCZ-treated and TCZ-untreated MAS patient groups. Table 1 shows the clinical characteristics of patient data at the time of MAS diagnosis.

**Table 1:** Demographic and clinical features of patients with macrophage activation syndrome

	Tocilizumab			Historic cohort		
	Full-blown	Possible	All	Full-blown	Possible	All
Number	2	10	12	10	8	18
Sex, no.						
Female	1	7	8	3	5	8
Male	1	3	4	7	3	10
Age, median (IQR)	6 (3–9)	8.5 (7–14.75)	8.5 (7–12.75)	5 (1.75–6.75)	8 (3.5–9.5)	5.5 (2–8.25)
Fever (%, n)	50 (1/2)	50 (5/10)*	50 (6/12)**	90 (9/10)	100 (8/8)*	94.4 (17/18)**
Hepatosplenomegaly (%, n)	0 (0/2)	30 (3/10)	25 (3/12)	40 (4/10)	37.5 (3/8)	38.9 (7/18)
Lymphadenopathy (%, n)	0 (0/2)	20 (2/10)	16.7 (2/12)	30 (3/10)	37.5 (3/8)	33.3 (6/18)
CNS (%, n)	0 (0/2)	0 (0/10)	0 (0/12)	0 (0/10)	0 (0/8)	0 (0/18)

*CNS* Central nervous system, * = *p* < 0.05; ** = *p* < 0.01;

### Statistical analysis

Within-group comparison was analyzed using Mann–Whitney test or Fisher’s exact test. *P* < 0.05 was considered statistically significant.

## Results

### TCZ modifies the clinical and laboratory features of s-JIA-associated MAS

Among the 12 TCZ-treated patients, only two were diagnosed with full-blown MAS and the remaining 10 were diagnosed with possible MAS. Conversely, among 18 TCZ-untreated patients, 10 were diagnosed with full-blown MAS and the remaining eight were diagnosed with possible MAS.

Table 1 shows the demographic and clinical features of the patients with MAS. In both the TCZ-treated patients and the TCZ-untreated patients, no significant differences with respect to age and sex were observed. The TCZ-treated patients at the time of MAS were significantly less likely to develop fever than the TCZ-untreated patients (Table 1) (possible MAS: *p* < 0.05; MAS All *p* < 0.01). In contrast, no significant differences were noted in other clinical features including hepatosplenomegaly and lymphadenopathy.

**Fig. 1:**
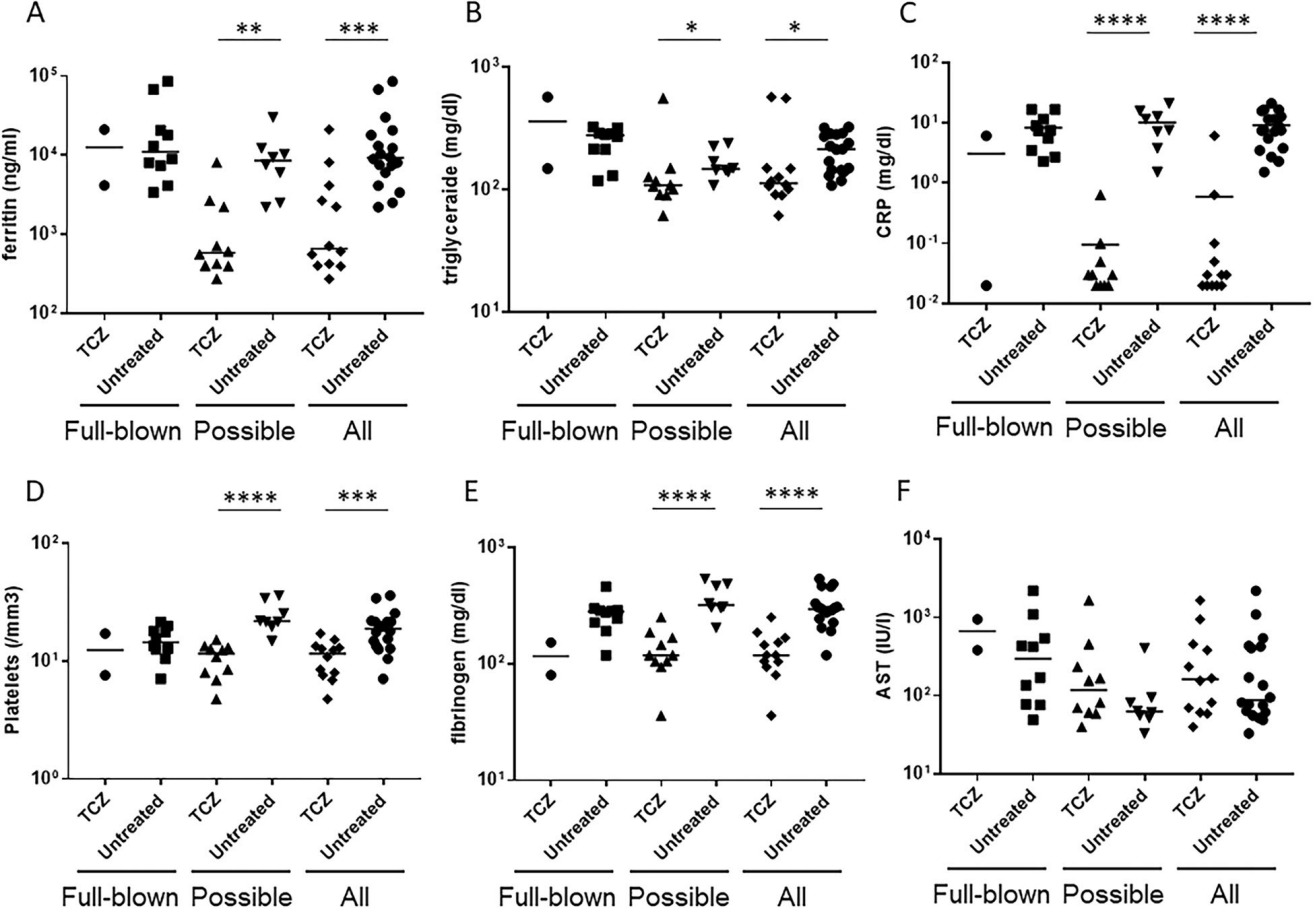
Inflammatory markers are modified in patients with systemic juvenile idiopathic arthritis associated macrophage activation syndrome while treated with tocilizumab, compared to the untreated patients Plots show **a**) serum ferritin levels, **b**) serum TG levels **c**) serum CRP levels, **d**) platelets count, **e**) plasma fibrinogen levels, and **f**) serum AST levels. Plots show median values. * = *p* < 0.05; ** = *p* < 0.01; *** = *p* < 0.001; **** = *p* < 0.0001.

Table 2 shows the laboratory findings at the time of MAS diagnosis in patients who developed MAS while treated with TCZ. Although statistical analysis could not be done because of the limited number of patients with full-blown MAS while treated with TCZ, the laboratory findings in patients with full-blown MAS while treated with TCZ seemed to be similarly severe compared with the TCZ-untreated patients (Table 2 and Fig. 1). In contrast, patients with possible MAS receiving TCZ had significantly lower serum ferritin levels than the TCZ-untreated patients (587 ng/ml vs 8518 ng/ml; *p* < 0.01) (Table 2 and Fig. 1a). Furthermore, patients with possible MAS receiving TCZ had significantly lower serum triglyceride (TG) levels (108 mg/dl vs 148 mg/dl; *p* < 0.05) (Table 2 and Fig. 1b) and serum C reactive protein (CRP) levels (0.03 mg/dl vs 9.6 mg/dl; *p* < 0.0001) than the TCZ-untreated patients (Table 2 and Fig. 1c). All patients with MAS (full-blown + possible MAS) receiving TCZ also had significantly lower serum ferritin levels (664 ng/ml vs 9235 ng/ml; *p* < 0.001) (Table 2 and Fig. 1a), serum TG levels (113 mg/dl vs 214 mg/dl; p < 0.05) (Table 2 and Fig. 1b), and serum CRP levels (0.03 mg/dl vs 7.6 mg/dl; *p* < 0.0001) than the TCZ-untreated patients (Table 2 and Fig. 1c). Furthermore, patients with possible MAS receiving TCZ also had significantly lower platelet counts (11.7 × 10^4^/mm^3^ vs 21.9 × 10^4^/mm^3^; *p* < 0.0001) (Table 2 and Fig. 1d) and lower fibrinogen levels (118 mg/dl vs 319 mg/dl; p < 0.0001) (Table 2 and Fig. 1e) than the TCZ-untreated patients. Patients with possible MAS receiving TCZ also had higher serum aspartic aminotransferase (AST) levels than the TCZ-untreated patients (119 IU/l vs 63 IU/l), but it was not statistically significant (Table 2 and Fig. 1f). All patients with MAS receiving TCZ also had significantly lower platelet counts (11.7 × 10^4^/mm^3^ vs 18.9 × 10^4^/mm^3^; *p* < 0.001) (Table 2 and Fig. 1d) and lower fibrinogen levels (118 mg/dl vs 294 mg/dl; *p* < 0.0001) (Table 2 and Fig. 1e) than the TCZ-untreated patients. All patients with MAS receiving TCZ also had higher serum AST levels than the TCZ-untreated patients (161 IU/l vs 89 IU/l), but it was not statistically significant (Table 2 and Fig. 1f). Taken together, we found significant differences in the key laboratory parameters associated with MAS in TCZ-treated patients.

**Table 2:** Laboratory findings of patients with macrophage activation syndrome

	Tocilizumab			Historic cohort		
	Full-blown (*n* = 2)	Possible (*n* = 10)	All (*n* = 12)	Full-blown (n = 10)	Possible (*n* = 8)	All (*n* = 18)
Ferritin (ng/ml)	12,645	587**	664***	11,035	8518**	9235***
	4155–21,135	402–2330	410–3785	6585–32,644	3390–11,833	5559–18,710
Platelets (× 10^4^/mm^3^)	12.4	11.7****	11.7***	14.5	21.9****	18.9***
	7.6–17.2	7.7–13.1	7.7–13.3	12.1–18.5	20.2–32.2	13.3–21.8
AST (IU/l)	666	119	161	296	63	89
	382–949	61–290	63–438	78–681	53–92	61–425
TG (mg/dl)	362	108*	113*	279	148*	214*
	149–574	91–132	94–150	192–298	141–213	143–285
Fibrinogen (mg/dl)	116	118****	118****	281	319****	294****
	80–152	102–172	97–163	217–292	302–482	240–362
CRP (mg/dl)	3.1	0.03****	0.03****	7.5	9.6****	7.6****
	0.0–6.1	0.0–0.6	0.0–6.1	3.3–12.9	4.7–15.1	3.7–13.6

Median values and IQR were shown. * = *p* < 0.05; ** = *p* < 0.01; *** = *p* < 0.001; **** = *p* < 0.0001; *AST* Aspartic aminotransferase, *TG* Triglyceride, *CRP* C reactive protein

### Validation of 2016 ACR/EULAR classification criteria of MAS complicating s-JIA while treated with TCZ upon MAS diagnosis

For patients with full-blown MAS treated with TCZ, these criteria classified as MAS 50% of patient events (1/2) collected in this study that had been clinically diagnosed as MAS (Table 3). This result compared with the TCZ-untreated patients, of whom 90% (9/10) were classified as MAS based on the derived criteria. For patients with possible MAS treated with TCZ, only 20% (2/10) could be classified as having MAS, lower than the historical cohort (75%, 6/8). For all patients with MAS (definite + possible MAS) treated with TCZ, only 25% (3/12) could be classified as having MAS, which was significantly lower than the TCZ-untreated patients (83.3%, 15/18, *p* < 0.01). TCZ-treated patients failed to fulfill the classification criteria due to an absence of fever or insufficient ferritin elevation (Table 3).

**Table 3:** Application of 2016 MAS classification criteria to patients with MAS treated with tocilizumab

	Tocilizumab			Historic cohort		
	Full-blown	Possible	All	Full-blown	Possible	All
Number with sufficient data	2	10	12	10	8	18
MAS (%, n)	50 (1/2)	20 (2/10)	25 (3/12)	90 (9/10)	75 (6/8)	83 (15/18)
Reasons missing (n)	Afebrile [1]	Afebrile [4]		Afebrile [1]	Other markers [2]	
		Ferritin [5]				

*MAS* Macrophage activation syndrome

## Discussion

Inflammatory cytokines including IL-1β, IL-6, and IL-18 play pathogenic roles in the disease processes of s-JIA [1]. Since the introduction of biologic agents, most notably targeting IL-1 and IL-6, treatment of s-JIA has dramatically developed. IL-6 plays an important role as an inflammatory mediator in the pathogenesis of s-JIA [10]. Serum IL-6 levels in patients with s-JIA correlate with the extent and severity of joint involvement, fever patterns, growth retardation, and osteoporosis [10]. The clinical use of TCZ had striking and long-lasting effects on s-JIA, even in patients with severe disease that was refractory to other therapies [11]. Despite the efficacy of TCZ treatment, patients with s-JIA still have a risk to develop MAS [3, 4, 7, 8]. The rates of MAS complications in s-JIA patients while treated with TCZ were 1.8–6.4 per 100 patients, which were similar to those reported in the patients not treated with biologic agents [7, 8, 12].

In this study, we analyzed whether there was any difference in the clinical and laboratory features of MAS in TCZ-treated patients. The data showed that there were significant alterations in the MAS parameters in TCZ-treated patients. In agreement with our previous case series and recent systematic review [3, 4], TCZ-treated patients were less likely to be febrile and had lower ferritin levels, CRP levels, platelet counts, fibrinogen levels, and TG levels than the historical cohort. These findings indicate that patients with MAS while treated with TCZ may have unconventional symptoms.

In this study, TCZ-treated patients were less likely to be febrile and had lower ferritin and CRP levels. IL-6 is a *representative cytokine to* induce the acute-phase response in the pathogenesis of s-JIA [10]. Therefore, modification of these clinical manifestations by TCZ might be reasonable. In contrast, interestingly, thrombocytopenia and hypofibrinogenemia became more pronounced with TCZ treatment. The reason for these modifications of laboratory findings is still unknown. One possibility is the suppressive effect by TCZ for the IL-6 mediated biosynthesis of fibrinogen in hepatocytes and IL-6 induced thrombopoiesis through thrombopoietin. Another possibility might be the side effect of TCZ itself. Some previous reports showed that s-JIA TCZ-treated patients developed thrombocytopenia [7, 13]. The other possibility is that TCZ might affect the disease process of s-JIA-associated MAS. Furthermore, TCZ masks the clinical symptoms of MAS including fever and elevation of ferritin and CRP levels. This modification might delay the recognition of MAS.

In this study, we validated the classification criteria for MAS in patients with MAS while treated with TCZ and found that the MAS classification criteria were less likely to classify the patients diagnosed with MAS while treated with TCZ due to an absence of fever or insufficient ferritin elevation, compared with the TCZ-untreated patients. The laboratory findings in patients with full-blown MAS while treated with TCZ seemed to be similarly severe compared with the untreated patients. However, one patient with full-blown MAS while treated with TCZ was afebrile even in the period of full-blown MAS. Furthermore, in this study, eight out of 18 patients in the untreated patients were diagnosed with possible MAS, whereas 10 out of 12 patients with MAS while treated with TCZ were diagnosed with possible MAS. For patients with possible MAS treated with TCZ, only 20% were classified as having MAS, lower than the untreated patients (75%). These findings indicate that TCZ could modify clinical manifestations and key laboratory findings of MAS; thus, these differences limit the applicability of these criteria. When evaluating s-JIA patients while treated with TCZ, care must be taken to not underdiagnose MAS based on MAS classification criteria.

Recent studies revealed some biomarkers including IL-18, CXCL9, neopterin and soluble tumor necrosis factor receptor type II might be useful for the prediction of the development of MAS and the diagnosis of the transition from active phase of s-JIA to MAS [14–21]. Recent studies revealed that high levels of free IL-18 (that is, IL-18 not bound to IL-18 binding protein) increases the risk of developing MAS [15, 22]. We previously reported that serum IL-18 levels were markedly elevated in patients with MAS while treated with TCZ and their levels positively correlated with the measures of disease activity [3]. Further larger studies are desired to develop the new criteria for the diagnosis of MAS while treated with TCZ using other parameters, including IL-18, whose serum levels are not modified by TCZ.

This study has some limitations. First, this study had a small sample size and was a retrospective analysis of a multicenter gathered cohort. Second, it was limited by the use of physician diagnosis to MAS because of an absence of gold standard for the diagnosis of MAS. Third, the suspicion for MAS might be higher in patients treated with TCZ compared to those not treated with TCZ, because the diagnosis of s-JIA were established in patients treated with TCZ. Further larger prospective studies might add value to this study.

## Conclusions

In conclusion, TCZ could modify clinical and laboratory features of MAS. When evaluating s-JIA patients while receiving TCZ, it is not applicable to use 2016 s-JIA associated MAS criteria for these group of patients. Care must be taken to not underdiagnose MAS based on the MAS classification criteria.

## Data Availability

The datasets used and/or analyzed during the current study are not publicly available for ethical reasons, as well as privacy reasons, but are available from the corresponding author on reasonable request.

## Abbreviations

AST: Aspartic aminotransferase
CRP: C reactive protein
IL: Interleukin
MAS: Macrophage activation syndrome
s-JIA: Systemic juvenile idiopathic arthritis
TCZ: Tocilizumab
TG: Triglyceride
